# SARS-CoV-2 Seroprevalence in Healthcare Workers of Kaunas Hospitals during the First Wave of the COVID-19 Pandemic

**DOI:** 10.3390/medicina57020148

**Published:** 2021-02-06

**Authors:** Laura Pereckaitė, Asta Dambrauskienė, Daiva Urbonienė, Saulius Sadauskas, Kęstutis Petrikonis, Albinas Naudžiūnas, Astra Vitkauskienė

**Affiliations:** 1 Department of Laboratory Medicine, The Hospital of Lithuanian University of Health Sciences Kauno Klinikos, 50161 Kaunas, Lithuania; dambrauskienea@gmail.com (A.D.); daiva.urboniene@kaunoklinikos.lt (D.U.); astra.vitkauskiene@kaunoklinikos.lt (A.V.); 2 Department of Internal Medicine, Kaunas Hospital of Lithuanian University of Health Sciences, 47144 Kaunas, Lithuania; Saulius.Sadauskas@lsmuni.lt (S.S.); albinas.naudziunas@lsmuni.lt (A.N.); 3 Department of Neurology, The Hospital of Lithuanian University of Health Sciences Kauno Klinikos, 50161 Kaunas, Lithuania; kestutis.petrikonis@lsmuni.lt

**Keywords:** COVID-19, SARS-CoV-2 seroprevalence, healthcare workers, ELISA, lateral flow immunoassay

## Abstract

*Background and objective*: Serologic testing is a useful additional method for the diagnosis of COVID-19. It is also used for population-based seroepidemiological studies. The objective of the study was to determine SARS-CoV-2 seroprevalence in healthcare workers of Kaunas hospitals and to compare two methods for specific SARS-CoV-2 antibody testing. *Materials and Methods*: A total of 432 healthcare workers in Kaunas hospitals were enrolled in this study. Each participant filled a questionnaire including questions about their demographics, contact with suspected or confirmed COVID-19, acute respiratory symptoms, and whether they contacted their general practitioner, could not come to work, or had to be hospitalized. Capillary blood was used to test for SARS-CoV-2 specific immunoglobulin G (IgG) and immunoglobulin M (IgM) a lateral flow immunoassay. Serum samples were used to test for specific IgG and IgA class immunoglobulins using semiquantitative enzyme-linked immunosorbent assay (ELISA) method. *Results*: 24.77% of study participants had direct contact with a suspected or confirmed case of COVID-19. A total of 64.81% of studied individuals had at least one symptom representing acute respiratory infection, compatible with COVID-19. Lateral flow immunoassay detected SARS-CoV-2 specific IgG class immunoglobulins in 1.16% of the tested group. Fever, cough, dyspnea, nausea, diarrhea, headache, conjunctivitis, muscle pain, and loss of smell and taste predominated in the anti-SARS-CoV-2 IgG-positive group. Using ELISA, specific IgG were detected in 1.32% of the tested samples. Diarrhea, loss of appetite, and loss of smell and taste sensations were the most predominant symptoms in anti-SARS-CoV-2 IgG-positive group. The positive percent agreement of the two testing methods was 50%, and negative percent agreement was 99.66%. *Conclusions*: 1.16% of tested healthcare workers of Kaunas hospitals were anti-SARS-CoV-2 IgG-positive. The negative percent agreement of the lateral flow immunoassay and ELISA exceeded 99%.

## 1. Introduction

Since its discovery in China at the end of 2019, the novel coronavirus, or severe acute respiratory syndrome coronavirus 2 (SARS-CoV-2), rapidly spread around the world, causing tens of millions of infection (COVID-19) cases [1]. In Lithuania, the first cases of COVID-19 infection were reported in March of 2020. Since then, a total of more than 160 thousand of COVID-19 cases are confirmed. In Kaunas, during the study period, from June to September of 2020, the number of unique SARS-CoV-2 positive cases grew from 102 to 521 [2].

SARS-CoV-2 is noted for its long incubation period, lower virulence, and high contagiousness; therefore, it usually causes asymptomatic or mild infection, which is effectively spread by unidentified or asymptomatic virus carriers [3,4]. SARS-CoV-2 is primarily transmitted by respiratory droplets, and might infect nasal, conjunctival, or oral mucosa upon inhalation or direct as well as indirect contact with infectious particles [3]. According to research data, SARS-CoV-2 seroprevalence is higher among healthcare workers compared to the general population [4,5]. Due to regular and direct contact with patients and colleagues, healthcare workers are at higher risk to get infected by SARS-CoV-2. Furthermore, medical workers with asymptomatic infection might cause most of the viral spread cases in health care environments [4,5].

In order to effectively control the spread of SARS-CoV-2 in community and health care facilities, rapid and accurate COVID-19 testing, as well as rigorous infection prevention and control practices, are indispensable [5,6]. So far, the main diagnostic test for COVID-19 remains reverse transcriptase polymerase chain reaction (RT-PCR), which detects SARS-CoV-2 ribonucleic acid (RNA) in respiratory secretion and saliva specimens. However, this diagnostic method is most useful during the acute phase of COVID-19. When testing asymptomatic persons, RT-PCR has lower sensitivity and is prone to false-negative results [7]. That is where serologic testing might find its place, as it helps determine present and past infection with SARS-CoV-2 [8,9]. Specific immunoglobulin M (IgM) class antibodies against this virus are usually detected in 5 to 12 days since the onset of infection, depending on its severity. Specific antibodies of immunoglobulin G (IgG) and immunoglobulin A (IgA) classes are usually detected later in the disease course, with a median time of 14 days. The kinetics of antibodies directed against the nucleocapsid and spike proteins are similar, although the immune response against nucleocapsid is detected earlier [9]. Moreover, serologic testing not only complements RT-PCR results for the diagnosis of COVID-19 but is also used for population-based seroepidemiological studies to determine SARS-CoV-2 seroprevalence [10]. The presence of antibodies in a certain community represents overall population exposure and may help predict the susceptibility to infection both of the members of the community and the representative population. SARS-CoV-2 seroprevalence in healthcare workers also helps to approximate possible transmission of the virus to patients and other staff [11].

We aimed to determine SARS-CoV-2 seroprevalence in healthcare workers of Kaunas hospitals during the first wave of the COVID-19 pandemic and to compare two methods for specific antibody testing.

## 2. Materials and Methods

Since the beginning of the COVID-19 pandemic in March until September, a total of approximately 150 SARS-CoV-2 infected persons, both patients and staff, were identified in two major hospitals of Kaunas. The infection with this virus was confirmed using RT-PCR at the Microbiology Laboratory of Hospital of Lithuanian University of Health Sciences Kauno Klinikos (HLUHS KK) Department of Laboratory Medicine. At HLUHS KK, most of the infected persons were patients of the Department of Emergency medicine. Kaunas Hospital of Lithuanian University of Health Sciences (KH LUHS), on the other hand, had a designated department for the management of the SARS-CoV-2 infected patients. Infection prevention and control measures in both hospitals, where surveyed respondents worked, were implemented according to the guidance documents provided by the European Centre for Disease Prevention and Control (ECDC). Physical distancing, hand and respiratory hygiene, and proper use of personal protective equipment (PPE) were the main means to minimize the risk of transmission of COVID-19. Other important approaches included visitor restriction rules, separation of patients with fever and respiratory symptoms in the emergency and primary care settings, isolation of SARS-CoV-2 positive patients, prompt risk assessment after patient or staff encounter with a confirmed COVID-19 case, and extensive testing and isolation of risky contacts [12].

The object of this prospective study was to test healthcare workers who had contact with patients having acute respiratory symptoms at Kaunas hospitals, including the HLUHS KK and KH LUHS. This study design was approved by the Kaunas Regional Biomedical Research Ethics Committee on 15 June 2020 (certificate No BE-2-75). Participants of the study were invited via e-mail, and they arrived to take part from 15 June to 21 September 2020.

A total of 432 healthcare workers in Kaunas hospitals were enrolled in this study. 85.42% (*n* = 369) of them were female. The mean age of all participants was 43.44 years (ranging from 21 to 74 years old, standard deviation ±12.57 years). Fourteen people (3.24%) were 65 years and older.

Each participant, consenting to be rolled into the study, was asked to fill a questionnaire including questions about their gender, age, the department they work at, smoking habits, contact with patients with suspected or confirmed COVID-19, acute respiratory symptoms, and whether they contacted their general practitioner, could not come to work or had to be hospitalized due to these symptoms. This questionnaire was made according to recommendations of the World Health Organization (WHO) regarding population-based seroepidemiological studies [13].

Capillary and venous blood samples were drawn at Procedure room of HLUHS KK Department of Laboratory Medicine. Finger capillary blood was used to test for specific IgG and IgM against SARS-CoV-2 using a lateral flow immunochromatographic assay “AMP Rapid Test SARS-CoV-2 IgG/IgM” (AMEDA Labordiagnostik GmbH, Graz, Austria) during the visit of the participant. In this test, a whole blood sample mixed with diluent migrates through the capillaries of the test membrane. If SARS-CoV-2 specific antibodies are present, they form immune complexes with recombinant SARS-CoV-2 antigens conjugated to colloid gold particles. These immune complexes are then caught by fixed anti-human IgG or anti-human IgM antibodies, and a respective (IgG or IgM) region of the test develops a red-colored band. Results of the test were determined visually using binary interpretation (negative or positive) and were only valid if a red band in the control region was clearly visible [14].

The analytical characteristics of the lateral flow immunoassay “AMP Rapid Test SARS-CoV-2 IgG/IgM” (AMEDA Labordiagnostik GmbH, Graz, Austria) are as follows. For the IgM test, the manufacturer reports the sensitivity of 95.7% and specificity of 97.3%. For the IgG test, sensitivity and specificity are lower—91.8% and 96.4%, respectively [14].

Serum separated from the venous blood sample was used to test for specific IgG and IgA against SARS-CoV-2 using semiquantitative enzyme-linked immunosorbent assay (ELISA) “Anti-SARS-CoV-2 ELISA IgG/IgA” (EUROIMMUN AG, Lübeck, Germany). In this kit, the wells of the microtitration plate are covered with domain S1 of the SARS-CoV-2 spike protein expressed recombinantly in human cells. ELISA tests were performed in accordance with manufacturer’s instructions at Microbiology Laboratory of HLUHS KK Department of Laboratory Medicine. For each sample, ratios of the diluted serum optical density and calibrator optical density were calculated and compared to the cut-off values provided by the manufacturer. Samples with a ratio of 1.1 were interpreted as positive for anti-SARS-CoV-2 IgG or IgA antibodies. Samples with a ratio of <0.8 were interpreted as negative for anti-SARS-CoV-2 IgG or IgA antibodies. Ratios of 0.8 to <1.1 yielded borderline results. Borderline results were not considered positive for the analysis [15,16].

The sensitivity of the ELISA diagnostic kit “Anti-SARS-CoV-2 ELISA IgG/IgA” (EUROIMMUN AG, Lübeck, Germany) is described considering the time passed from the onset of the symptoms or positive result of direct detection. For the IgG ELISA, the manufacturer reports the sensitivity of only 43.7% during the first 10 d since the onset of the symptoms, and 94.4% after 10 days. The specificity of the test in both cases is 99.6%. For the IgA ELISA, the sensitivity during the first 10 d of the disease is 88.2%, and 96.9% after 10 d. The specificity of this test is 98.3% [15,16].

Questionnaire data, lateral flow immunoassay, and ELISA test results were analyzed using “IBM SPSS” software. The mean, range, and standard deviation were calculated for quantitative data. Age between groups was compared using the Mann-Whitney U test. Pearson’s Chi-square, Fisher tests, were used to analyze qualitative data. Statistically significant findings were assumed with *p* < 0.05.

## 3. Results

A total of 412 (95.14%) study participants worked at HLUHS KK, and 20 (4.63%) at KH LUHS. Also, 39.12% (*n* = 169) were physicians, 41.44% (*n* = 179) were nurses, 12.04% (*n* = 52) were assistance personnel, and 6.71% (*n* = 29) were other staff. The remainder of the respondents did not disclose their working place (*n* = 3). Most of the study participants—16.67% (*n* = 72) worked in therapeutic profile departments, 14.12% (*n* = 61)—in intensive care units, 13.89% (*n* = 60)—in emergency medicine, 13.43% (*n* = 58)—in surgical profile departments. Others included pediatric and neonatal profile departments—8.56% (*n* = 37), diagnostic and rehabilitation profile departments—each 7.41% (*n* = 32), gynecology and obstetrics departments—5.79% (*n* = 25). Less than 1.5% of study participants worked at ambulatory departments (*n* = 6), pediatric intensive care units (*n* = 5), HLUHS KK Blood Center (*n* = 4), KH LUHS Infectious Diseases Department (*n* = 4), hematology and oncology profile departments (*n* = 3), and others (*n* = 7). The remainder of the respondents did not disclose their working place (*n* = 26).

A total of 12.96% (*n* = 56) of participants indicated that they smoked during the study period, and 24.77% (*n* = 107) of studied healthcare workers stated that they have had contact with a suspected or confirmed case of COVID-19 since the beginning of the pandemic. However, the private or professional circumstances of the possible contagion were not clarified.

A total of 64.81% (*n* = 280) of participants noted that during the period of three months they had at least one symptom representing acute respiratory infection, including COVID-19. The most commonly specified symptoms were as following: headache—40.05% (*n* = 173), fatigue—35.65% (*n* = 154), sore throat—27.08% (*n* = 117), runny nose—21.06% (*n* = 91), cough—18.06% (*n* = 78), diarrhea—9.72% (*n* = 42), and joint pain—9.26% (*n* = 40). Only 9.29% (*n* = 25) of participants who have had symptoms consulted their general practitioner, and 7.50% (*n* = 21) of these could not come to work. None of those who have had any symptoms had to be hospitalized. Specific IgG class antibodies against SARS-CoV-2 were found in five (1.16%) study participants using the rapid lateral flow immunoassay. Two of them (0.46%) also had specific IgM class antibodies against SARS-CoV-2.

The ELISA method was used to confirm the seroprevalence testing results of the lateral flow immunoassay. Serum samples of the first 302 study participants were tested. Ratio values equal to 1.1 and higher were interpreted as positive for both IgG and IgA ELISA testing. Borderline results were not interpreted as positive.

IgG antibodies against SARS-CoV-2 were found in 4 (1.32%) cases. ELISA ratio values ranged from 1.373 to 6.945, with a mean of 3.34, and median of 2.53. IgA antibodies were found in 11 (3.64%) samples. ELISA ratio values ranged from 1.150 to 7.042, with a mean of 2.72, and a median of 1.87.

Three (0.99%) of tested participants had both IgG and IgA specific antibodies against SARS-CoV-2. IgG ELISA ratios varied from 2.482 to 6.945 (mean ratio 4.000). IgA ELISA ratios varied from 1.150 to 7.042 (mean ratio 3.200).

There were only two cases when both ELISA and lateral flow immunoassay detected IgG anti-SARS-CoV-2. ELISA IgG ratio values in these cases were 2.573 and 6.945. In the cases where anti-SARS-CoV-2 IgG was not detected using lateral flow immunoassay, ELISA IgG ratio values were 1.373 and 2.482.

A detailed review of the questionnaire answers of the participants with specific antibodies of class IgG, IgM, and IgA against SARS-CoV-2 is shown in Table 1.

To summarize the data presented in Table 1, 60% of anti-SARS-CoV-2 IgG-positive individuals, detected using rapid lateral flow immunoassay, were female, however, there was not a statistically significant difference in gender between IgG-positive and IgG-negative participants. The mean age of IgG-positive individuals did not statistically significantly differ between gender groups. A total of 80% of participants worked at HLUHS KK. Most of them were physicians and nurses working in adult therapy, pediatric, neonatal therapy, surgery and emergency medicine departments. Only 20% of IgG-positive individuals smoked at the time of the survey. It is important to note that all anti-SARS-CoV-2 IgG-positive individuals reported their direct contact with a suspected or confirmed case of COVID-19. Fever (60%), cough (60%), dyspnea (40%), nausea (40%), diarrhea (40%), headache (100%), conjunctivitis (20%), muscle pain (60%), and loss of smell and taste sensations (40% each) of statistical significance dominate the anti-SARS-CoV-2 IgG-positive group compared to the IgG-negative group. Also, 60% of IgG-positive individuals consulted their general practitioner, and 80% of them could not show up at work due to these symptoms, which was also a statistically significant finding comparing to the IgG-negative group.

In the anti-SARS-CoV-2 IgG ELISA positive individuals, the distribution of females and males was equal; however, their age was significantly lower compared to the IgG-negative group. Also, 75% of them worked at HLUHS KK, and 75% of anti-SARS-CoV-2 IgG-positive individuals, detected using the ELISA testing method, knew about a previous contact with a suspected or confirmed case of COVID-19. The most predominant symptoms in this group were diarrhea (75%), loss of appetite (25%), and loss of smell and taste sensations (50% each). A total of 25% of the IgG-positive individuals had to refer to their general practitioner and could not come to work due to these symptoms, and none of them had to be hospitalized.

Among the anti-SARS-CoV-2 IgA ELISA positive participants, 72.73% were female. The mean age in this group was also statistically significantly lower than in the IgA-negative group. A total of 81.82% of these individuals worked at HLUHS KK. More than half of them reported that they had contact with a suspected or confirmed case of COVID-19. The most statistically significantly predominant symptoms in this group were diarrhea (36.36%), and loss of smell and taste sensations (18.18% each). Only one (9.09%) of these IgA-positive healthcare workers consulted their general practitioner and could not show up at work due to their symptoms.

The agreement study was performed in 302 paired samples of the study participants in order to compare SARS-CoV-2-specific IgG class antibodies detection in capillary blood via lateral flow immunoassay (“AMP Rapid Test SARS-CoV-2 IgG/IgM”, AMEDA Labordiagnostik GmbH, Graz, Austria) with antibodies detection in sera tested by ELISA method (“Anti-SARS-CoV-2 ELISA IgG”, EUROIMMUN AG, Lübeck, Germany). The data of this comparison study are shown in Table 2.

The positive percent agreement (PPA) of these two testing methods was 50%, and the negative percent agreement (NPA) was 99.66%.

## 4. Discussion

The data of this study revealed that a quarter (24.77%) of questioned healthcare workers have had contact with a suspected or confirmed case of COVID-19 infection. Two-thirds (64.81%) of study participants also had acute respiratory symptoms resembling COVID-19, namely, headache, fatigue, sore throat, runny nose, cough, diarrhea, and joint pain. However, only one-tenth (9.29%) of them consulted their general practitioner due to these symptoms.

Specific SARS-CoV-2 IgG class antibodies using the rapid lateral flow immunoassay were detected in 1.16% of tested medical workers of Kaunas hospitals. They worked in adult therapeutical, pediatric, and neonatal therapeutical and surgical profile departments, and contacted the patient ill with COVID-19. Fever, cough, dyspnea, nausea, diarrhea, headache, conjunctivitis, muscle pain, as well as the loss of smell and taste were statistically significantly more prevalent in those individuals compared to the IgG-negative group. Among IgG ELISA positive cases, symptoms of diarrhea, loss of appetite, and loss of smell and taste sensations were significantly more predominant.

During the international research initiated by Great Ormond Street Institute of Child Health (UK), about 300 healthcare workers routinely contacting pediatric patients with fever and acute respiratory symptoms were tested at Vilnius University Hospital Santaros Klinikos. According to the study data, only 2 (0.66%) of tested hospital workers had specific IgG class antibodies against the novel coronavirus. They also had COVID-19 confirmed using RT-PCR before the study [11]. Similar results were found in a German study where 16.1% of healthcare workers had confirmed contact, and 39.2% with a suspected case of COVID-19. Even so, 45.2% developed at least one acute respiratory symptom, and only 0.92% (*n* = 2) of study participants had detectable IgG antibodies against SARS-CoV-2 [10]. In a large Danish study, 4.04% of healthcare workers were tested positive for IgG and/or IgM specific antibodies against the novel coronavirus. The highest seroprevalence was among medical students (14.97%), paramedics (9.95%), and nursing assistants (4.65%). The lowest seroprevalence was found in laboratory specialists (1.93%). Furthermore, seroprevalence in men was statistically significantly higher than in women. During the recent 6 months, 52.8% of antibody-negative and 78.1% of specific antibody-positive participants had at least one symptom representing COVID-19. The most notable in the latter group was the loss of smell and taste sensations [17]. Another study conducted in the United Kingdom analyzed asymptomatic healthcare workers, and 2.4% of them had SARS-CoV-2 RNA detectable using RT-PCR. A total of 15.4% of the studied participants had specific antibodies against this virus. However, in a symptomatic group, seroprevalence was higher [18]. Differently to the findings of the studies reviewed above, 19.1% of healthcare workers had specific IgG class antibodies against SARS-CoV-2 in Sweden. Most of these statistically significantly cases had a fever and lost sensations of smell and taste. The frequency of sore throat was similar in specific antibody-positive and -negative groups [19].

We found that, despite quite a high self-reported rate of contact with suspected or confirmed COVID-19, only a small fraction of healthcare workers had developed specific antibodies against the novel coronavirus during the first wave of the pandemic. Our results indicate the proper and effective use of personal protective equipment and other infection prevention measures. They also represent the situation among medical workers of Kaunas hospitals during the period from June to September 2020, when the overall prevalence of COVID-19 was not high in Kaunas.

Since there is no standardization of the serological assays, seroprevalence differences between countries and tested cohorts within the countries complicate the interpretation of those data [11]. Discrepancies between different study data might also be due to different testing methods and diagnostic kits used in those studies. The differences in COVID-19 prevalence in tested populations as well as the different approaches to infection prevention and control also affect the results of the studies [11]. According to Beinortas et al., our results could also be associated with a low prevalence of SARS-CoV-2 in the community as well as the lower sensitivity of diagnostic tests in this setting [20]. As a matter of course, data found in our study is not final because population-based seroprevalence is constantly changing as the numbers of confirmed COVID-19 cases continue to grow.

The agreement study for the detection of SARS-CoV-2 specific IgG class antibodies in capillary blood by lateral flow immunoassay and the detection of them in sera via the ELISA method showed that PPA was 50%, but it is important to note that NPA between both tests reached 99.66%. Despite the fact that these measures are identical to those commonly used for sensitivity and specificity, in this case, PPA and NPA reflect the situation better because none of the compared methods are considered as a reference or diagnostic “gold standard” [21]. Furthermore, at the time of the survey, we were not aware of the true state of the study individuals, or whether they were tested via RT-PCR to confirm SARS-CoV-2 infection. According to the Food and Drug Administration (FDA), the clinical accuracy of the studied tests has to be validated using specimens from patients with microbiologically confirmed COVID-19 infection [22]. Therefore, due to the lack of validation and small sample size tested, we cannot conclude which diagnostic method performs better in this study. Further serological testing using a more sensitive method and investigation is needed in order to identify the factors causing discrepancies and low statistical agreement value.

Bastos et al., reviewing multiple serologic methods for detection of both IgG and IgM SARS-CoV-2 specific antibodies, summarized that the sensitivity of different lateral flow immunoassays varied between 30.7 and 100%. The overall sensitivity of the reviewed tests was 66% (95% CI, 49.3–79.3%). The sensitivity of ELISA tests was up to 84.3% (95% CI, 75.6–90.9). The specificity of lateral flow immunoassays and ELISA were similar, 96.6% and 97.6%, respectively. There was no statistically significant difference among these tests while detecting IgG and IgM class antibodies separately [8]. Another study comparing the performance of two commercially available lateral flow immunoassays and the ELISA testing method found their agreement to be up to 84.7–94.0%. However, the positive prognostic value of those two rapid tests was only 29.0–72.2% [5]. Mercado et al. compared the performance of nine immunochromatographic tests for the diagnosis of COVID-19. The IgG and IgM detection specificities of the AMP Rapid Test were accordingly 97% and 95.50%. However, this test showed very low sensitivity for IgG and IgM detection in asymptomatic individuals, only 18.42% and 28.95%, respectively. At 8–11 days after the onset of symptoms, the IgG and IgM detection sensitivities were marginally better—22.22% and 33.33%, with their peak at >11 days—79.16% and 70.83%, respectively [23]. Traugott et al., reviewing multiple different serological methods, determined specificities of Euroimmun ELISA IgG and IgA of 98% and 83%, respectively. The sensitivity of the Euroimmun ELISA IgA increased over the period of 1–5 to 6–10 days after the onset of symptoms from 30% to 84%, and sensitivity of Euroimmun ELISA IgG increased from 3.3% to 40%. Both testing methods reached their highest sensitivity of 100% after 11 days since the onset of symptoms [24]. In a bigger study, Nilsson et al., comparing six commercially available SARS-CoV-2 antibody assays, found that the Euroimmun ELISA IgG had a specificity of 96.2%, while the ELISA IgA diagnostic kit had significantly lower specificity of only 73.2%. To improve the latter, according to the epidemiological setting, the researchers suggest increasing the cut-off value to 4.0 in order to yield a specificity of more than 97.5%. The sensitivity of Euroimmun ELISA IgG was analyzed over the course of COVID-19 illness. From days 8–14 to 22–28, the sensitivity increased from 57% to 92%, reaching its maximum of 96% at >28 days after the onset of symptoms [25]. According to Nilsson et al. and the Euroimmun anti-SARS-CoV-2 ELISA IgA Instruction for use, due to its lower specificity, this assay is not recommended for the screening of asymptomatic individuals. It is better suited for monitoring the development of an immune response after direct detection of the virus. Hence, it can possibly explain our finding that anti-SARS-CoV-2 IgA seroprevalence is higher compared to IgG seroprevalence [16,25].

Although lateral flow immunoassays are inexpensive, simple to perform, and easily applied for point-of-care testing, various authors agree that their sensitivity is too low for the diagnosis of COVID-19. Using lateral flow immunoassay, 44–87% of RT-PCR confirmed patients would be falsely tested negative during the first week of COVID-19 illness. Even during the third week, up to 30% of SARS-CoV-2 positive cases might be undiagnosed [8].

As SARS-CoV-2 specific antibodies start to appear 5–7 days after infection, they are not a reliable marker for early acute infection diagnostics. The most important use of serologic testing so far is for population exposure testing and infection risk assessment at the individual level. However, the strength of immunity that antibodies confer is so far unclear [26]. Scientists note that milder infection rarely leads to developing strong and long-term immunity provided by specific antibodies against SARS-CoV-2, compared to the severe form of COVID-19, with the involvement of lower respiratory tract. It might be explained by the fact that lower viral load is neutralized by the innate immune system and does not trigger a specific immune response [5]. Furthermore, antibodies formed against different components of the virus have different neutralizing capacities, which plays a major role in preventing reinfection. According to research data, antibodies targeting the receptor-binding domain (RBD) and S2 region of the spike protein display the strongest neutralizing activity [9]. Finally, not only should humoral immunity, mediated by specific antibodies, be taken into account, but cellular immunity against viral infections plays an important part in this process, too. So far, molecular and immunological investigations of cellular immunity against SARS-CoV-2 are still complicated and not applied in routine settings [27].

## 5. Conclusions

In summary, 24.77% of tested healthcare workers in Kaunas hospitals stated that they had direct contact with a suspected or confirmed case of COVID-19 since the beginning of the pandemic. A total of 64.81% of study participants had at least one symptom representing acute respiratory infection, compatible with COVID-19. Lateral flow immunoassay detected SARS-CoV-2 specific IgG class immunoglobulins in only 1.16% of the tested sample. Using ELISA, specific IgG were detected in 1.32% of the tested samples. Fever, cough, dyspnea, nausea, diarrhea, headache, conjunctivitis, muscle pain, and loss of smell and taste were most commonly associated with anti-SARS-CoV-2 IgG seropositivity. The negative percent agreement for lateral flow immunoassay and ELISA exceeded 99%. Despite these impressive results, further investigations are required in order to draw reliable conclusions about performance characteristics of diagnostic tests for SARS-CoV-2 specific antibodies.

## Figures and Tables

**Table 1:** Review of the participants with SARS-CoV-2 specific antibodies of immunoglobulin classes G, M, and A (IgG, IgM, and IgA) detected using lateral flow immunoassay and enzyme-linked immunosorbent assay (ELISA).

Characteristic	Lateral Flow Immunoassay in the Capillary Clood (*N* = 432)		ELISA in the Venous Blood Serum (*N* = 302)	
	IgG	IgM	IgG	IgA
	*n* (%)	*n* (%)	*n* (%)	*n* (%)
	5 (1.16)	2 (0.46)	4 (1.32)	11 (3.64)
**Gender**				
• male	2 (40)	0 (0)	2 (50)	3 (27.27)
• female	3 (60)	2 (100)	2 (50)	8 (72.73)
**Age, years**				
• mean	48.60	57	30.25 ^1^	34.36 ^1^
• range	26–59	55–59	23–47	25–55
• standard deviation	±13.39	±2.83	±11.24	±9.95
**Hospital**				
• HLUHS KK	4 (80)	2 (100)	3 (75)	9 (81.82)
• KH LUHS	1 (20)	0 (0)	1 (25)	2 (18.18)
**Profile of the department**				
• pediatric and neonatal therapy, surgery, and emergency medicine	2 (40)	0 (0)	1 (25)	3 (27.27)
• adult therapy	2 (40)	1 (50) ^2^	1 (25)	2 (18.18)
• infectious diseases	0 (0)	0 (0)	0 (0)	1 (9.09)
• adult intensive care units	0 (0)	0 (0)	2 (50)	4 (36.36)
• other	1 (20)	1 (50) ^2^	0 (0)	1 (9.09)
**Position**				
• physicians	2 (40)	0 (0)	2 (50)	5 (45.45)
• nurses	2 (40)	1 (50)	2 (50)	6 (54.54)
• other	1 (20)	1 (50)	0 (0)	0 (0)
**Smoking**	1 (20)	1 (50)	0 (0)	2 (18.18)
Previous contact with a suspected or confirmed case of COVID-19	5 (100) ^1^	2 (100)	3 (75)	6 (54.54)
**Signs and symptoms**				
• fever	3 (60) ^2^	2 (100) ^2^	1 (25)	1 (9.09)
• sore throat	1 (20)	0 (0)	1 (25)	2 (18.18)
• runny nose	1 (20)	0 (0)	1 (25)	3 (27.27)
• cough	3 (60) ^1^	1 (50)	1 (25)	3 (27.27)
• dyspnea	2 (40) ^1^	2 (100) ^1^	0 (0)	0 (0)
• nausea	2 (40) ^1^	1 (50)1	1 (25)	1 (9.09)
• diarrhea	2 (40) ^1^	0 (0)	3 (75) ^2^	4 (36.36) ^1^
• headache	5 (100) ^1^	2 (100)	3 (75)	6 (54.54)
• conjunctivitis	1 (20) ^1^	0 (0)	0 (0)	0 (0)
• muscle pain	3 (60) ^2^	1 (50) ^1^	1 (25)	2 (18.18)
• loss of appetite	1 (20)	0 (0)	1 (25) ^1^	1 (9.09)
• loss of smell	2 (40) ^2^	0 (0)	2 (50) ^2^	2 (18.18) ^2^
• loss of taste	2(40) ^2^	0 (0)	2 (50) ^2^	2 (18.18) ^2^
• fatigue	3 (60)	1 (50)	1 (25)	3 (27.27)
Due to these symptoms consulted with a general practitioner	3 (60) ^2^	1 (50) ^1^	1 (25)	1 (9.09)
Due to these symptoms could not come to work	4 (80) ^2^	2 (100) ^2^	1 (25)	1 (9.09)
Due to these symptoms had to be hospitalized	0 (0)	0 (0)	0 (0)	0 (0)

^1^ Statistically significant, *p* < 0.05. ^2^ Statistically significant, *p* < 0.001.

**Table 2:** Comparison of the detection of SARS-CoV-2 specific immunoglobulin G (IgG) class antibodies using lateral flow immunoassay and enzyme-linked immunosorbent assay (ELISA).

		Lateral Flow Immunoassay (Capillary Blood)		
		Positive, *n*	Negative, *n*	Total, *N*
ELISA (Serum)	Positive, *n*	2	2	4
	Negative, *n*	1	297	298
	Total, *N*	3	299	302

## Data Availability

The data presented in this study are available on request from the corresponding author.
